# Entanglement rates and haulout abundance trends of Steller (*Eumetopias jubatus*) and California (*Zalophus californianus*) sea lions on the north coast of Washington state

**DOI:** 10.1371/journal.pone.0237178

**Published:** 2020-08-25

**Authors:** Elizabeth Marina Allyn, Jonathan Joseph Scordino

**Affiliations:** Marine Mammal Program, Makah Fisheries Management, Makah Tribe, Neah Bay, Washington, United States of America; NOAA Fisheries, UNITED STATES

## Abstract

Entanglements affect marine mammal species around the globe, and for some, those impacts are great enough to cause population declines. This study aimed to document rates and causes of entanglement and trends in local haulout abundance for Steller and California sea lions on the north coast of Washington from 2010–2018. We conducted small boat surveys to count sea lions and document entangled individuals. Rates of entanglement and entangling material occurrence were compared with records of stranded individuals on the Washington and Oregon coast and with packing bands recorded during beach debris surveys. The rate of entanglement for California sea lions was 2.13%, almost entirely composed of adult males, with a peak rate during June and July potentially due to some entangled individuals not migrating to their breeding grounds. For Steller sea lions, the rate of entanglement was 0.41%, composed of 77% adults (32.4% male, 63.3% female), 17.1% juveniles, 5.9% unknown age, and no pups. Steller sea lions exhibited a 7.9% ± 3.2 rate of increase in abundance at the study haulouts, which was similar to that seen in California sea lions (7.8% ± 4.2); both increases were greater than the population growth rates observed range-wide despite high rates of entanglement. Most entanglements for both species were classified as packing bands, followed by entanglement scars. Salmon flashers were also prevalent and only occurred from June–September during the local ocean salmon troll fishery. Packing band occurrence in beach debris surveys correlated with packing band entanglements observed on haulouts. However, no packing band entanglements were observed in the stranding record and the rate of stranded animals exhibiting evidence of entanglement was lower than expected, indicating that entanglement survival is higher than previously assumed. Future studies tracking individual entanglement outcomes are needed to develop effective, targeted management strategies.

## Introduction

The prevalence of man-made marine debris is of global concern and has been gaining attention from media, researchers, and the public in recent decades as the impacts to marine life become better understood [1–5]. Many marine organisms are affected by marine debris and other man-made materials through entanglement. Instances of entanglement have been recorded for at least 32 species of marine mammals globally [4], and for some, like the northern fur seal (*Callorhinus ursinus*) and the endangered Hawaiian monk seal (*Monachus schauinslandi*), entanglement was thought to have contributed to population declines [6–8]. For pinnipeds specifically, entanglement has been documented for more than half of the existing species [2,4,9]. In this study, entanglement is defined as the presence of entangling materials attached to an animal’s body, including materials that are looped around the appendages, torso, or neck (e.g. netting or packing bands) and instances where materials are internally or externally embedded (e.g. hooking injuries).

The mechanisms by which an animal becomes entangled are almost as varied as the entangling materials themselves. Entangling materials can come from terrestrial and marine pollution, and from derelict and active fishing gear. Any materials that form loops that can ensnare or sharp edges that can embed pose an entanglement risk. The mechanism of entanglement can often be determined by identifying the entangling material. Packing bands and rubber bands are likely encountered passively as debris, while monofilament line, rope, and net fragments can be a sign of either passive encounters with derelict gear or a sign of interaction with an active fishing set. Salmon flashers and other hook and line setups are likely encountered as actively fished gear and are evidence of fishery depredation behaviors, which cause harm both to the entangled animal and to the fisher’s catch [10,11]. Otariids are especially curious of novel objects, and can become entangled in materials while attempting to explore or play with them [12,13]. The frequency and nature of entangling interactions with marine debris might be governed by ocean currents, upwelling patterns, and marine traffic patterns, while interactions with active or derelict fishing gear are driven by fishing effort, gear types, and prey distribution [9,14–17]. Each type of entangling material poses different challenges and opportunities for mitigation, so identifying the source of entangling materials is crucial to building effective and targeted prevention plans.

The objective of this study was to characterize the rates and causes of entanglement in Steller (*Eumetopias jubatus*) and California (*Zalophus californianus*) sea lions in northern Washington state and to evaluate the relationship between local entanglement rates and haulout abundance trends. We described temporal trends in entanglement occurrence and determined the most commonly observed entangling materials. Based on previous studies, we expected to mainly see entanglements caused by packing bands and netting [1,4,9,18–20]. We expected little change in annual entanglement occurrence but anticipated that there would be a peak in entanglements observed in the summer months due to these being the peak months for recreational and commercial fishing effort. We also compared entanglement rates with beach debris survey data to discern patterns in entanglement occurrence due to material availability, and with the stranding record to briefly explore the impacts of entanglement on health and survival. Understanding the patterns behind entanglement occurrence will enable the development of more targeted prevention and response efforts and a more accurate understanding of the impacts of entanglement on local populations.

## Methods

### Data collection

The National Marine Fisheries Service reviewed and approved our research methodologies and granted Marine Mammal Protection Act research permits 14326, 13430, and 19430. We also obtained Special Use Permits from the United States Fish and Wildlife Service for all land-based survey activities conducted on haulouts within the Flattery Rocks National Wildlife Refuge.

**Fig 1:**
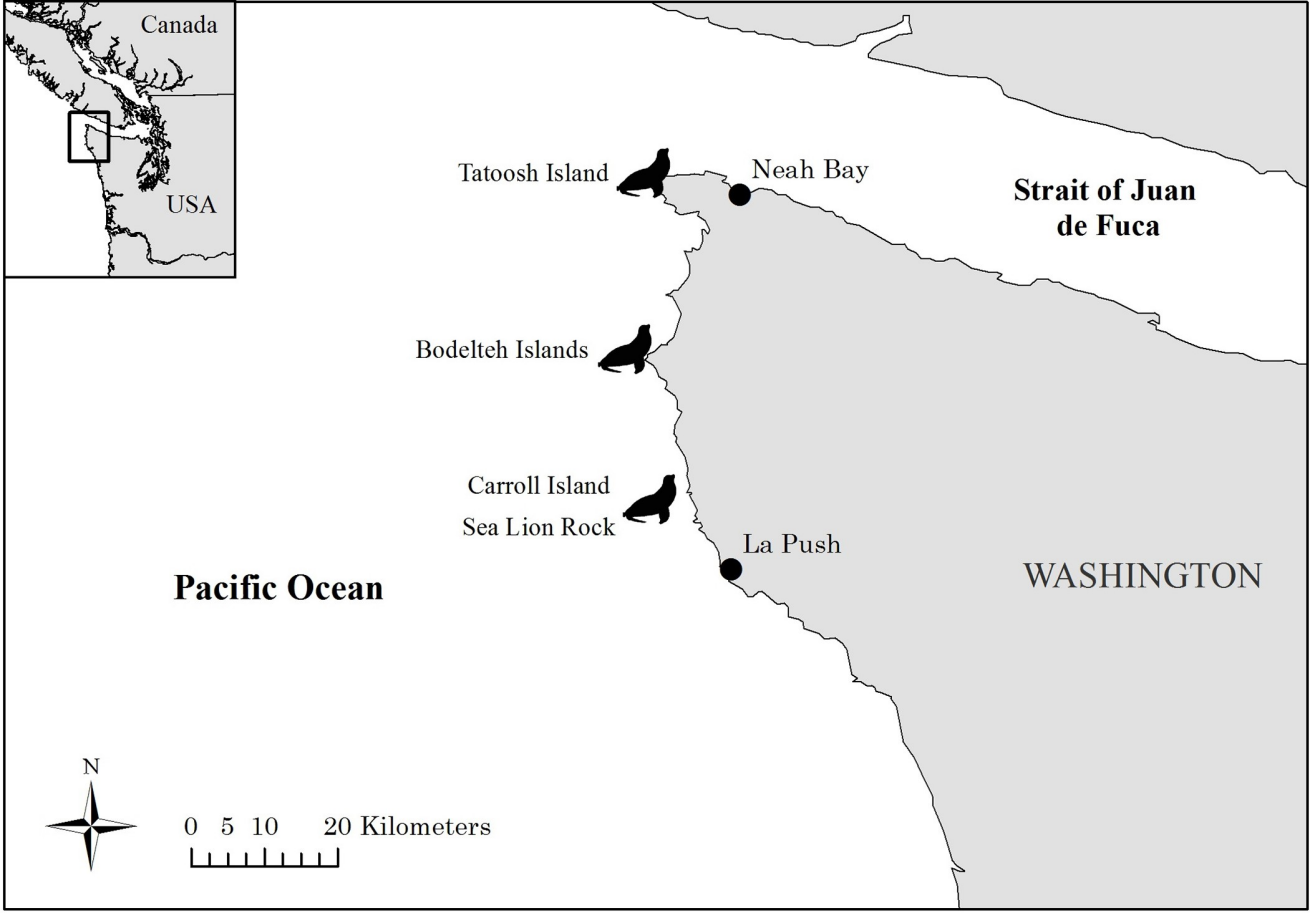
Map of the four major Steller and California sea lion haulout complexes surveyed for entangled individuals: Tatoosh Island, the Bodelteh Islands, Carroll Island, and Sea Lion Rock.

Observations of hauled out Steller and California sea lions were carried out from small boats along the north coast of Washington from 2010–2018 focusing on four major haulout complexes: Tatoosh Island (48.39° N, 124.74° W), the Bodelteh Islands (48.18° N, 124.76° W), Sea Lion Rock (47.99° N, 124.73° W), and Carroll Island (48.00° N, 124.72° W) (Fig 1). Occasionally, researchers were landed on haulouts to conduct these surveys. Surveys were conducted year-round with more effort from late spring through early fall due to availability of survey days with suitable weather and sea conditions. Surveys often did not include all haulouts due to logistical challenges such as sea conditions and daylight, but only complete survey days where all four major haulouts were visited were included in haulout abundance calculations. During surveys, we counted actively entangled individuals and individuals showing evidence of past entanglement (e.g. scarring) and counted the total abundance of the two sea lion species at each haulout. We attempted to photograph all entangled sea lions and those that appeared entangled with a digital SLR camera with a 100–400 mm lens for later assessment. Entangled individuals encountered along the survey route in locations other than the four major haulout complexes were excluded from entanglement rate calculations due to the lack of reliable and regular total counts of hauled individuals but were still photographed to identify the source and nature of the injury. Entanglement and count data are publicly available through Mendeley Data [21].

### Haulout abundance trends

We calculated an average rate of haulout abundance change for California and Steller sea lions on the northern Washington coast for 2010–2018 using surveys of four major haulout complexes (Fig 1). Our survey effort was greatest during the summer and early fall when sea conditions were most predictable (Table 1). To avoid disproportionately representing times of the year when more survey effort was conducted, trends in local haulout abundance were calculated using a multi-step process. First, for each species we pooled the counts from the four major haulout complexes on days when all four haulout complexes were visited (a ‘complete survey day’). Next, we averaged all complete survey days within each month of the 9-year study period. Last, we took the mean of the monthly averages within each year for an annual estimate of the average abundance of Steller and California sea lions using the four major haulout complexes. The observed changes in annual counts were calculated for each year using the formula rt=ln(Nt+1Nt) where *r*_*t*_ is the realized per capita rate of haulout abundance change, *t* is the year, and *N* is the average count for the year. The annual rates of change were then averaged over all study years to produce the overall average rate of change in haulout counts for each species. We excluded 2018 data from the analysis because there were no survey days that covered all four haulout sites after June, potentially biasing the counts by not including the full range of seasonal variation (Table 1).

**Table 1:** The number of sea lion haulout surveys in northern Washington conducted in each month of the study period 2010–2018 with the number of complete surveys where all four major haulout complexes were visited in parentheses. Note that no complete surveys were conducted after June in 2018.

Month														
		1	2	3	4	5	6	7	8	9	10	11	12	Total
**Year**	2010		1	1	1	3(1)	2	3(1)	5(3)	8(2)	2	2(1)		28(8)
	2011	1	2(1)	2	4(1)	6(3)	5(2)	4(2)	6(2)	3(2)	4(1)	3(1)		40(15)
	2012		2	2(1)	2(1)	3(2)	5(4)	8(1)	4(2)	5(2)	2(1)	3(1)	2(1)	38(16)
	2013	2(1)	1(1)	2(1)	2(1)	3(1)	4(3)	3(2)	3(2)	3(1)	3	2		28(13)
	2014				2	2(1)	3(2)	4(2)	4(1)	4(3)	2			21(9)
	2015	3(2)	2(1)	1	3	2	2	4(1)	5(2)	4(1)	1	1		28(7)
	2016	1(1)		4	1	5(3)	1(1)	4(2)	4(2)	3(3)	3(1)		1(1)	27(14)
	2017	1(1)	2(1)	1			3(3)	1	4(3)	3(1)	1(1)			16(10)
	2018		1	3(1)	2(1)	1(1)	3(2)	3	3	3	1			20(5)
	**Total**	**8(5)**	**11(4)**	**16(3)**	**17(4)**	**25(12)**	**28(17)**	**34(11)**	**38(17)**	**36(15)**	**19(4)**	**11(3)**	**3(2)**	**246(97)**

### Entanglement rates

We calculated an average entanglement rate for California and Steller sea lions for the northern Washington coast using counts of entangled sea lions and total haulout complex counts. Our survey effort was greatest during the summer and early fall when sea conditions were most predictable (Table 1). In order to ensure that our calculated annual entanglement rates were representative of the year, and not disproportionately representing time periods when we had more surveys, we calculated average yearly entanglement rates using a multistep process. Counts of the total number of individuals hauled out and counts of entangled individuals, including both active and inactive entanglements recorded from photographs and survey notes, were pooled across haulout complexes within survey days, and an entanglement rate was calculated for each survey day by dividing the total number of entangled individuals by the total count. Average entanglement rates were then calculated for each month of the nine-year study period. The mean rates for each month of the study were then averaged across years for each month and across months for each year to discern seasonal and annual patterns, which were analyzed using single-factor ANOVA and Tukey-Kramer post-hoc tests. An overall average entanglement rate was calculated for each species by taking the average of the monthly mean entanglement rates. We conducted a literature review to catalog published entanglement rates for California and Steller sea lions along with other otariid species to provide a comparison to our calculated rates.

### Photo analysis

We assessed photographs of sea lions with evidence of entanglement to determine if the entanglement was active or inactive, identify the entangling material, and record the age and sex of the entangled individual. Entangled individuals were assigned to demographic groups by age as adult, juvenile, pup, or unknown, and by sex for adults based on a number of physical characteristics, including body size and shape, whisker length, and presence of secondary sexual features. The proportions of entangled individuals in each sex and age class were calculated.

**Fig 2:**
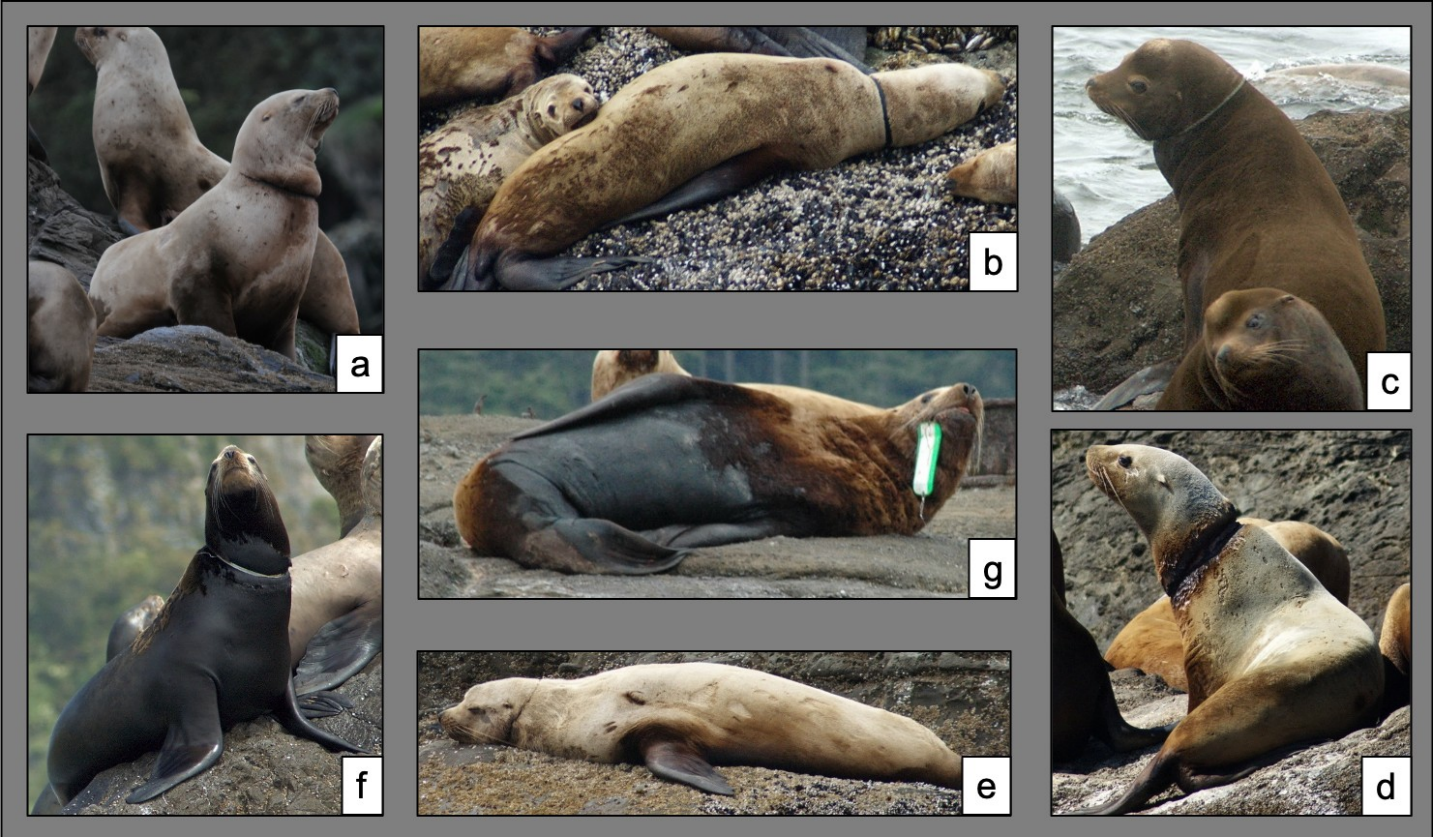
Example photographs of entangled Steller and California sea lions observed during small boat surveys of sea lion haulout complexes on the north coast of Washington from 2010–2018. Clockwise from the top left: a) Steller sea lion with an entanglement scar, b) Steller sea lion with a rubber band entanglement, c) California sea lion with a rope entanglement, d) Steller sea lion with a severe entanglement wound where the material is unidentifiable, e) Steller sea lion entangled in monofilament line, f) California sea lion entangled by a packing band, and g) Steller sea lion with a salmon flasher entanglement.

Entangling materials were identified to one of nine categories: packing band, salmon flasher, rubber band, monofilament line, hook and line, netting, rope, scar, or unknown (Fig 2). Salmon flashers are plastic or metal attractants attached to a line with a 60 – 200cm leader ahead of the lure or baited hook. The hook is often swallowed leaving the flasher to dangle out of the mouth by the leader. The hook and line category included fishing lures (not attached to flashers) and longline gear, both of which are found hooked externally on entangled individuals. Rubber bands are thick black bands cut from truck tire inner tubes that are often used in crab fisheries to secure trap doors. Packing bands are thin plastic strips attached at the ends to form loops that are used to increase the integrity of containers generally made of cardboard. The netting category included both gillnets made of monofilament line and trawl netting made of nylon or synthetic lines. Monofilament lines are commonly used in recreational fisheries and for leaders in commercial salmon fisheries and were differentiated from gillnets by the absence of knotted webbing. Active entanglements where the material could not be identified were recorded as ‘Unknown’. Animals with evidence of a previous entanglement where no material was observed on the sea lion were recorded as ‘Scar’. The proportion of entanglements that were active or inactive and the proportion exhibiting each entangling material were summarized and reported over months and years to observe trends in material occurrence.

### Packing band analysis

Annual packing band entanglement occurrence was compared to data from beach debris surveys conducted by the Olympic Coast National Marine Sanctuary (OCNMS) to discern patterns in material availability in the environment. The year 2018 was excluded from analysis due to low sea lion survey effort after the month of June. OCNMS conducted 1,548 beach debris surveys in the Olympic Coast region from 2012–2017, covering 17 beaches in Washington State, from Roosevelt Beach (47.1770°N, 124.1972°W) to Wa’atch Beach (48.3441°N, 124.6792°W). Surveys were conducted by volunteers in an OCNMS citizen science program adhering to standardized debris monitoring procedures developed by NOAA’s Marine Debris Program [22]. The number of packing bands encountered each year in beach debris surveys was divided by the total number of surveys conducted in that year to correct for variation in survey effort. The annual proportion of entanglements caused by packing bands observed during surveys was analyzed for correlation with the number of packing bands per beach debris survey.

### Stranding analysis

The West Coast Marine Mammal Stranding Network, overseen by NOAA’s West Coast Regional Office Protected Resources Division, has recorded sightings of marine mammal strandings since the early 1980s. Network members recorded evidence of entanglement on examined stranded sea lions. Data on Steller and California sea lions that stranded dead on the Washington and Oregon coast from 2010–2018 were analyzed to determine the occurrence of stranded individuals bearing evidence of entanglement. Entanglements were assigned to three categories depending on the nature of the entanglement evidence: animals that stranded with the entangling material still present were marked as ‘Active’, animals with evidence of entanglement-related injuries without entangling material present were marked ‘Scar’, and animals showing probable but inconclusive evidence of entanglement were marked ‘Possible’. For active entanglements, the entangling material was determined using notes and comments accompanying the stranding record and assigned to one of the categories used during our live surveys (e.g. packing band, flasher). Entanglements marked ‘Possible’ were excluded from summary statistics due to inconsistencies in reporting suspicious lesions as potential entanglement evidence.

### Statistical analysis

**Fig 3:**
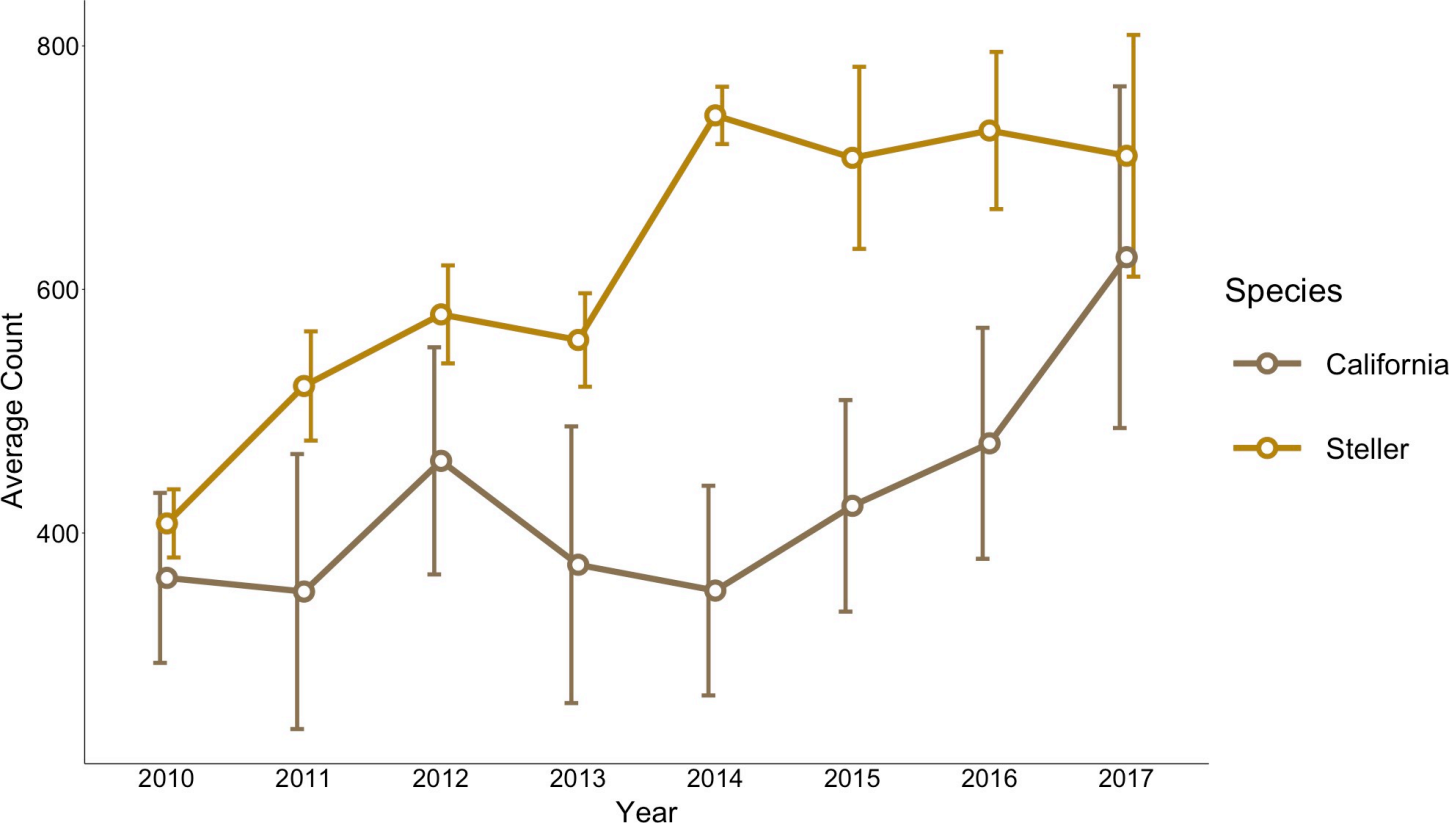
Trends in average annual counts of Steller and California sea lions present at four major haulout complexes on the north coast of Washington from 2010–2017.

**Fig 4:**
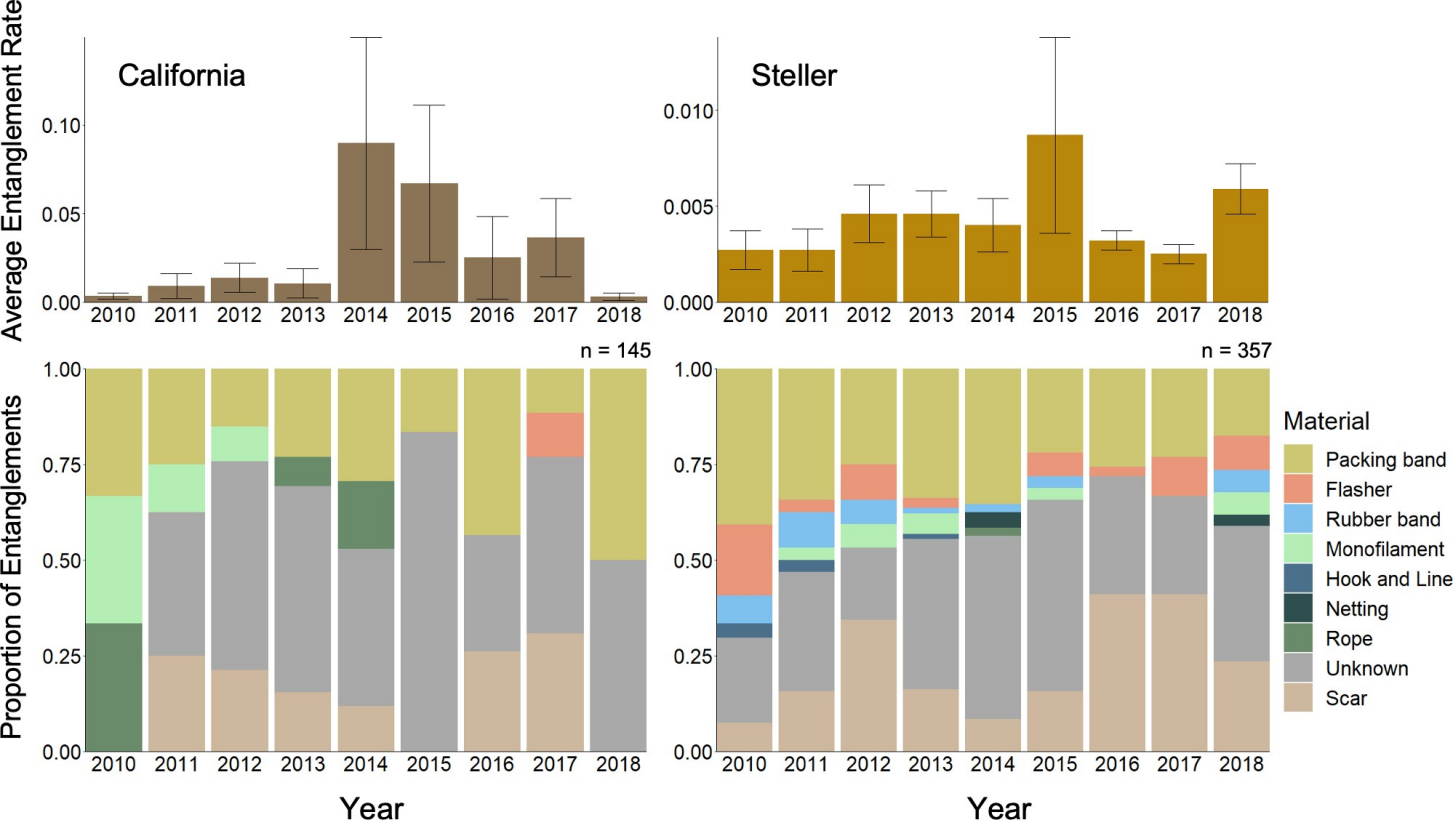
Average entanglement rates (expressed as entanglements per individual) and entangling material proportions for California and Steller sea lions in northern Washington from 2010–2018 by year. Entanglement rate calculations only included entangled individuals observed at one of four major haulout complexes. Entangling materials were only analyzed for individuals with photos of sufficient quality observed hauled out anywhere along the survey route.

**Fig 5:**
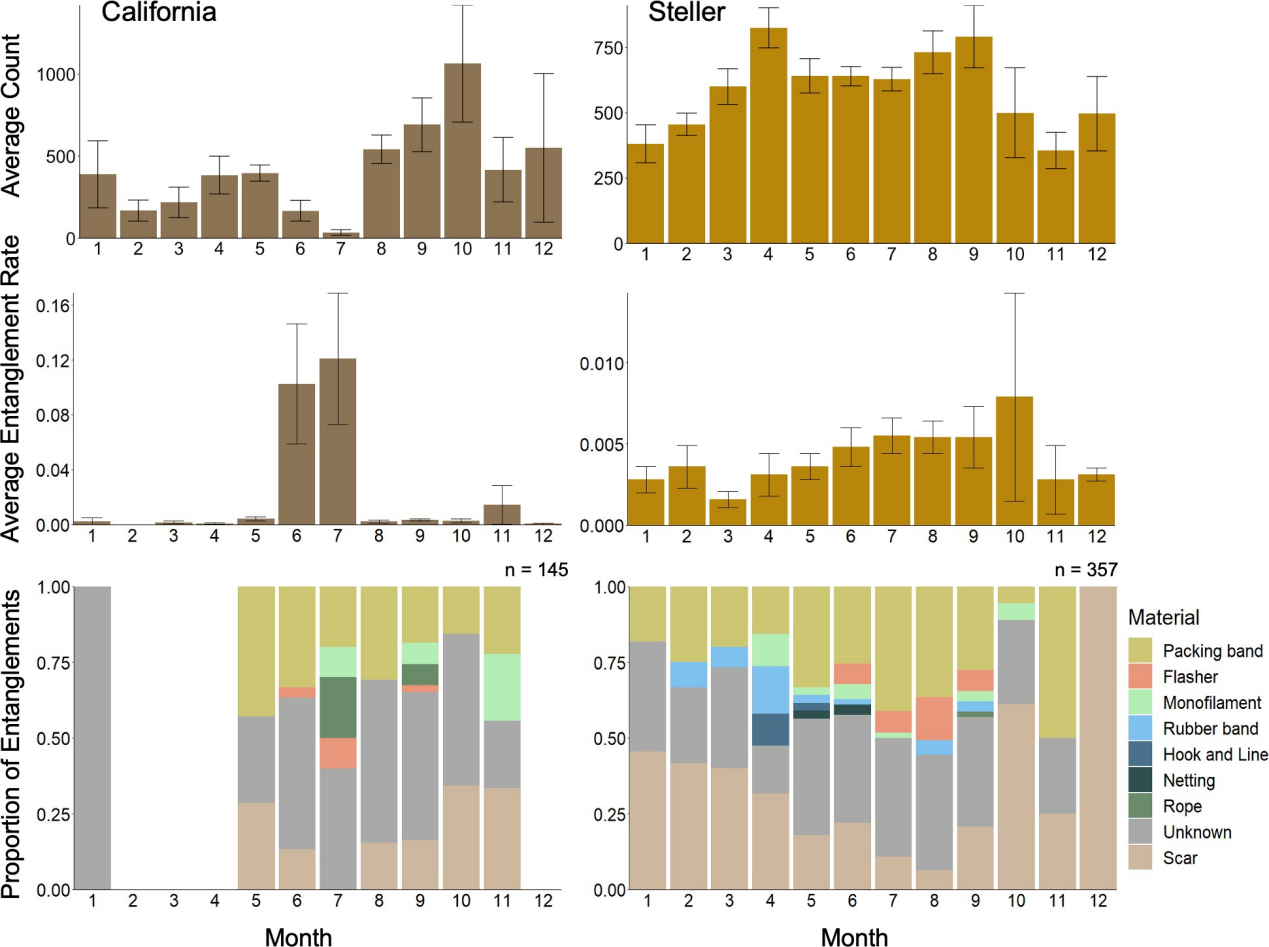
Average pooled counts at the four major haulouts, average entanglement rates (expressed as entanglements per individual), and entangling material proportions for California and Steller sea lions in northern Washington from 2010–2018 by month. Entanglement rate calculations only included entangled individuals observed at one of four major haulout complexes. Entangling materials were analyzed for any entangled individuals with photos of sufficient quality observed hauled out anywhere along the survey route.

**Fig 6:**
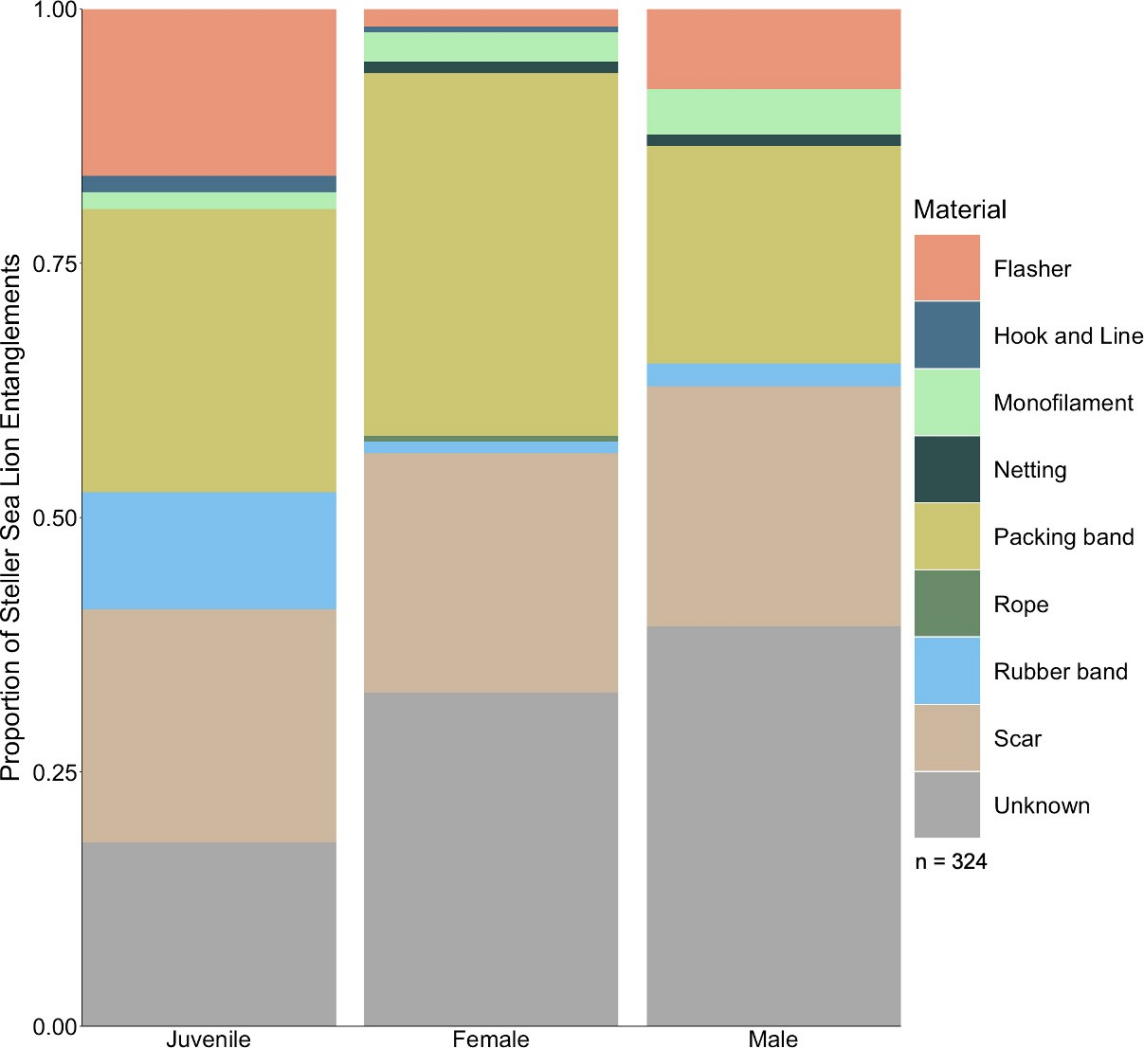
The proportion of entanglements caused by each material type for Steller sea lion juveniles (both sexes), adult females, and adult males in northern Washington, 2010–2018.

**Fig 7:**
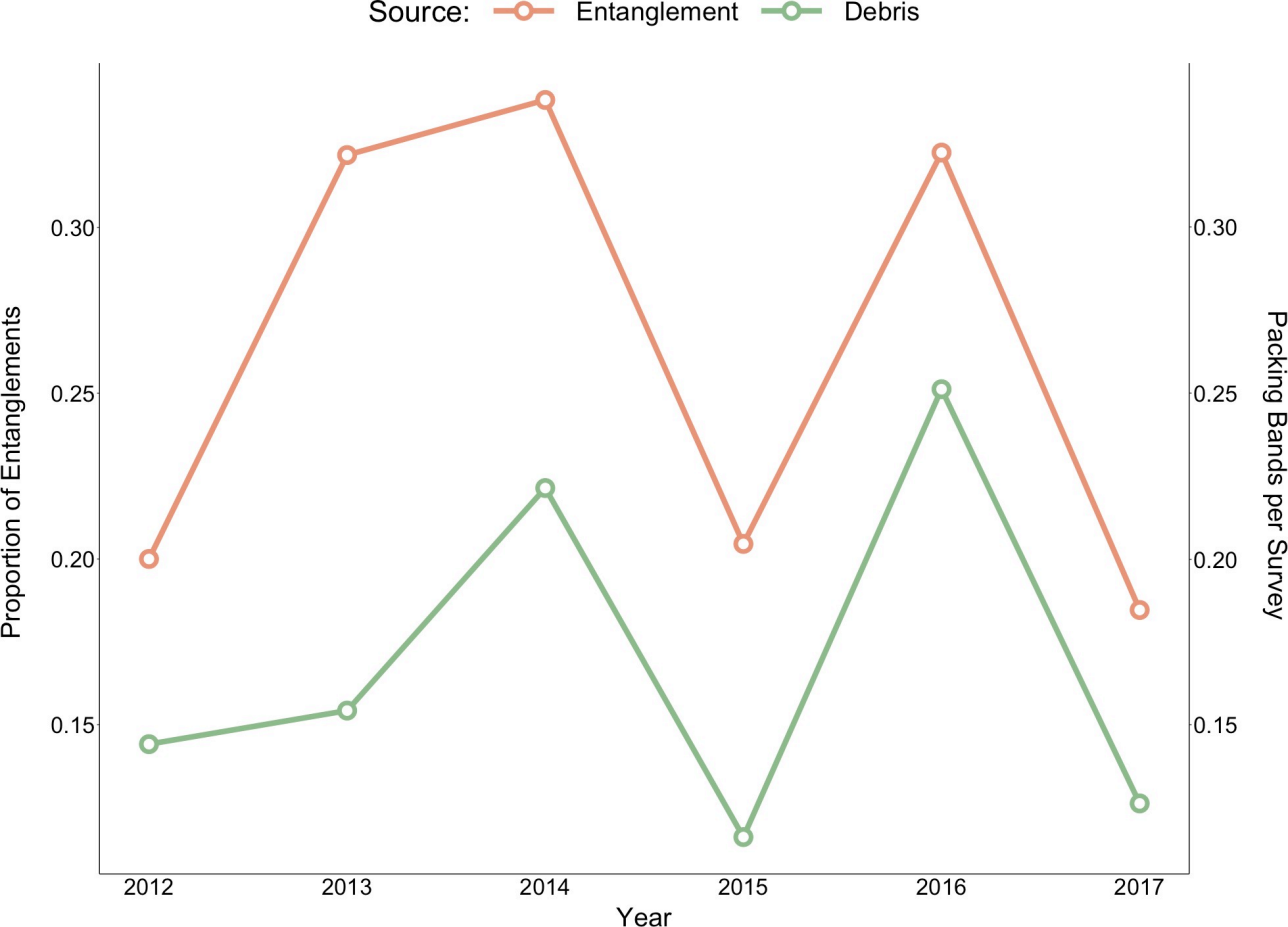
The proportion of entanglements caused by packing bands for sea lions at haulouts in northern Washington (primary axis) and the number of packing bands per survey recorded in beach debris surveys along the north Pacific coast of Washington conducted by the Olympic Coast National Marine Sanctuary (secondary axis).

All statistical analyses were conducted in Microsoft Excel. Figs 3–7 were created with R Statistical Program version 3.6.1 using ggplot2 [23,24].

## Results

### Haulout abundance trends

There were 97 survey days from 2010–2017 where counts were recorded at all four major haulout complexes (Table 1). The average annual rate of change of abundance at the haulout complexes in northern Washington for Steller sea lions was 7.9% ± 3.2 (95% CI), and for California sea lions was 7.8% ± 4.2 (95% CI; Fig 3).

### Entanglement rates

There were 648 observations of active and inactive entanglements in the survey area from 2010–2018, 611 (433 Steller, 178 California) of which were documented at the four major haulout complexes. The average overall entanglement rate for California sea lions was 2.13%, and for Steller sea lions was 0.41%. There were no annual or seasonal trends of statistical significance in entanglement rates for Steller or California sea lions (Fig 4). However, California sea lions experienced high rates of entanglement in 2014, and both species experienced somewhat elevated rates of entanglement in 2015. California sea lions also exhibited some seasonal variability with a peak in entanglement rates in the summer, coinciding with the lowest months for haulout abundance (Fig 5). While other months exhibited elevated rates of entanglement (November: 1.5%) or comparatively low average haulout counts (February: 168, March: 218), June and July were the only months to exhibit both low average haulout counts and high entanglement rates (June: 167, 10.2%; July: 35, 12.1%).

### Material analysis

There were 502 (357 Steller, 145 California) sightings of entanglements with photos of a quality sufficient for analysis. For Steller sea lions, inactive entanglements (scars) comprised 22.1% of all entanglements. Of the remaining active entanglements, only 55.4% (n = 154) were identifiable. The majority of identifiable entanglements were caused by packing bands (67.5%) and salmon flashers (13.6%). Other materials comprised less than 10% of identifiable entanglements: rubber bands (7.8%), monofilament line (6.5%), netting (1.9%), hook and line (1.9%), and rope (0.6%). For California sea lions, 80.0% of all entanglements were active, and 41.4% (n = 48) of active entanglements were identifiable. Packing bands made up the majority of entanglements (70.8%), followed by monofilament line (12.5%), rope (10.4%), and salmon flashers (6.3%). For both species, salmon flashers were only observed in the months of June–September coinciding with the local recreational and commercial ocean salmon troll fishery (Fig 5). In all cases where the entangling material could not be identified or was no longer present the entanglement scar or wound was located on the neck, indicating that those entanglements were caused by an encircling material, such as a packing band, rubber band, monofilament line, rope, or netting.

### Sex and age

For Steller sea lions both the sex and age could be identified for 74.5% of entanglements, and either the sex or the age could be identified for an additional 19.9% of the 357 Steller sea lion entanglement cases analyzed. The age composition of entangled Steller sea lions was 77% adults (32.4% male, 63.3% female), 17.1% juveniles, 5.9% unknown age, and no pups. For the most part, entangling materials were evenly distributed among sex and age classes, but 16.4% of entangled juveniles exhibited a flasher and 11.5% exhibited rubber bands, higher percentages than any other sex or age class grouping (Fig 6). The sex and age could be identified for 98.6% (n = 143) of entangled California sea lions, 142 of which were adult males, with one juvenile male. The single juvenile male was entangled in a packing band.

### Packing band analysis

Annual trends in the proportion of entanglements caused by packing bands from 2012–2017 positively correlated with the annual occurrence of packing bands observed during OCNMS beach debris surveys (Pearson’s R = 0.81; Fig 7).

### Stranding analysis

There were confirmed stranding records of 551 dead Steller sea lions and 1,048 dead California sea lions on the outer coast of Washington and Oregon from 2010–2018. The rate of dead strandings exhibiting evidence of entanglement (1.6% for Steller sea lions and 0.38% for California sea lions) was of a similar magnitude to the rate of entanglement among live sea lions observed on the haulouts (Table 2). All 4 entangled California sea lions that stranded dead were adult males. Of the 9 dead stranded entangled Steller sea lions, 7 were adults (4 females, 3 males), 1 juvenile, and 1 unknown. Of the 13 total entanglements observed, 5 were entangled in salmon flashers and other assorted hook and line gear. There was also a single Steller sea lion entangled in rope, and another exhibiting scars indicative of entanglement. The remaining 6 records did not have enough detail to determine the status of the entanglement or the entangling material. No sea lions stranded dead were recorded entangled in packing bands.

**Table 2:** Entanglement rates for Steller and California sea lions recorded dead in the Washington and Oregon coast stranding record and for sea lions observed alive on haulouts during surveys of northern Washington.

	Stranding Entanglement Rate	Haulout Entanglement Rate
*Steller sea lions*	1.63%	0.41%
*California sea lions*	0.38%	2.13%

## Discussion

Despite exhibiting high rates of entanglement, both California sea lions and Steller sea lions exhibited high rates of haulout abundance increase in northern Washington. The California sea lions in this study experienced the second highest entanglement rate for any otariid in the published literature and the highest otariid entanglement rate documented in the United States (Table 3). The observed rate of increase in local haulout abundance for California sea lions (7.8%) was greater than the estimated growth rate of the range-wide population in recent years as the population approached carrying capacity, and was similar to the maximum range-wide population growth estimate observed from 1975–2014 (7%) [25]. The entanglement rate observed in this study for Steller sea lions was almost double other published rates [18,26; Table 3], and the haulout abundance increase rate calculated for Steller sea lions in this study (7.9%) was more than double the growth rate observed by Pitcher et al. [27] and the National Marine Fisheries Service [28] using population estimates based on pup counts for the eastern distinct population segment of Steller sea lions (3.1%). While local haulout abundance trends alone cannot be used to make conclusions regarding the trajectory of the population as a whole, or the impact that entanglement might be having range-wide, it is important to note that use of the study area continues to increase despite such high entanglement rates, and that both populations as a whole are healthy and growing. Future studies that incorporate entanglement data from the whole range of each species could illuminate the full impact that entanglements may be having on the two species. Furthermore, a longer-termed study could detect delayed impacts of entanglements on local abundance trends that might have been outside the timeframe of this study.

**Table 3:** A review of pinniped entanglement rates in the published literature in ascending order of entanglement rate.

Year	Reference	Location	Species	Rate (%)
1988–1989	[20]	Channel Islands, CA	Cu	0.00
1997–2013	[29]	Bass Strait, Australia	App	0.002–0.019
1988–1997	[30]	Livingston Island, Antarctica	Ag	0.024
1996–2002	[31]	Bouvetøya	Ag	0.024–0.059
2001–2005	[1]	Point Reyes, CA	Zc	0.03
1982–1984	[32]	St Paul Island, AK	Cu	0.04^~^
1985	[26]	Aleutian Islands, AK	Ej	0.07
1978	[33]	Namibia & South Africa	Ap	0.11*
1977	[33]	Namibia & South Africa	Ap	0.12*
1979	[33]	Namibia & South Africa	Ap	0.12*
1991–1996	[34]	Marion Island, Australia	Ag & At	0.15
2006	[35]	Pribilof Islands, AK	Cu	0.17*
1983–1984	[20]	Channel Islands, CA	Zc	0.18
2005	[35]	Pribilof Islands, AK	Cu	0.18*
2006	[35]	St Paul Island, AK	Cu	0.20
1988–2000	[36]	Kangaroo Island, Australia	Nc	0.20
1988–1989	[20]	Channel Islands, CA	Zc	0.22
1985–1986	[20]	Channel Islands, CA	Cu	0.24
1996–1999	[34]	Marion Island, Australia	Ag & At	0.24
2001–2007	[18]	SEAK & northern BC	Ej	0.26
1985–1986	[20]	Channel Islands, CA	Zc	0.27
1986–1988	[20]	Channel Islands, CA	Zc	0.27
1986–1988	[20]	Channel Islands, CA	Cu	0.28
1988–1989	[37]	Bird Island, South Georgia	Ag	0.4
1989–2000	[36]	Kangaroo Island, Australia	Af	0.4
2010–2018	[21]	Northwest Coast, WA	Ej	0.43
1991–1995	[38]	Gulf of California, Mexico	Zc	0.49
1995–2005	[39]	Kaikoura, New Zealand	Af	0.6–2.84
1983	[40]	St Paul Island, AK	Cu	0.75*
1984	[41]	St Paul Island, AK	Cu	0.78*
2001–2002	[36]	Kangaroo Island, Australia	Af	0.9
2001	[36]	Kangaroo Island, Australia	Nc	1
2002	[36]	Kangaroo Island, Australia	Nc	1.3
1989–1991	[42]	Bass Strait, Australia	Apd	1.9
2010–2018	[21]	Northwest Coast, WA	Zc	2.86
1992	[16]	Los Islotes, Mexico	Zc	3.9–7.9
2000	[43]	Los Islotes, Mexico	Zc	8.75
1998	[43]	Los Islotes, Mexico	Zc	9.9
1992	[43]	Los Islotes, Mexico	Zc	10.4

Entanglement rates were calculated using many different methodologies based on many different data collection methods and are not meant to be directly comparable without caution. Species are listed using the first letters of their genus and species: Af—Arctocephalus forsteri, Ag–Arctocephalus gazella, Ap–Arctocephalus pusillus, Apd–Arctocephalus pusillus doriferus, App–Arctocephalus pusillus pusillus, At–Arctocephalus tropicalis, Cu–Callorhinus ursinus, Ej–Eumetopias jubatus, Nc–Neophoca cinerea, Zc–Zalophus californianus.

* Harvest data, only subadult males

~ Rookery data during breeding season

While the entanglement rates we observed were high, the low number of recorded mortalities from entanglement in the literature and in the local stranding record highlights our poor understanding of the effects of entanglement on sea lion health and survival. In the stranding record for the Washington and Oregon coast only 13 California and Steller sea lions were found dead with signs of entanglement from 2010–2018 out of 1,599 total strandings. The rate of dead stranded sea lions that exhibited evidence of entanglement (0.81%) was of a similar order of magnitude to the rate of live sea lions observed with signs of entanglement from survey effort (0.41% Steller, 2.13% California). In the literature there are also very few records of animals observed dead with signs of entanglement [19,44]. Since dead stranded animals are a subset of the mortality experienced by a population, it is logical that if entanglement always had a significant negative effect on the sea lion’s health and survival, the proportion of dead individuals with evidence of entanglement would be greater than for the live population at large. Since recorded mortality due to entanglement was lower than expected, it suggests that this was not the case.

The definition of serious injury developed and used by the National Oceanic and Atmospheric Administration (NOAA) is “an injury that will likely result in mortality” [45]. According to the guidelines, which categorize most entanglements as serious injuries, including “Ingestion of gear or hook” and “Gear constricted on any body part, or likely to become constricting as the animal grows”, most active entanglements observed in this study would be classified as serious injuries, with the exception of two Steller sea lions who exhibited hooks externally on the flank and side of the head [45]. In assessments by NOAA of data from 2010–2017, all entanglements categorized as serious injuries with descriptions similar to what we observed and that did not receive rehabilitation or disentanglement assistance were recorded as mortalities [46–57]. However, this study presents multiple lines of evidence refuting the idea that entanglement without intervention is always a death sentence for the affected individual. Studies on tagged subadult male northern fur seals on St. Paul Island, Alaska found that entangled individuals had a similar return rate the following year as the general harvest population, suggesting that entanglement, at least for the harvestable segment of the population, had little to no impact on short-term survival [41,58]. However, the probability of long-term survival might be largely dependent on the animal’s ability to shed the entangling material [41]. There are records of animals shedding entangling materials in the wild, including an adult female Antarctic fur seal (*Arctocephalus gazella*) that removed a tied loop of rope [59], a female Hawaiian monk seal with a nursing pup who freed herself from a tangle of monofilament and polypropylene line [60], nursing female northern fur seals who freed themselves from 200g trawl net fragments [61], multiple Hawaiian monk seals who seemed to entangle and disentangle themselves in beached netting [44], and several Steller sea lions, including a few branded individuals, observed shedding salmon flashers and one neck entanglement in Alaska (Alaska Department of Fish and Game, unpublished data; [49,52]). Likewise, while packing bands were the most common entangling material in all study years for both Steller and California sea lions from live observations, similar to what was seen in other studies in the North Pacific [18,20,35], not a single sea lion stranded dead on the Washington or Oregon coast from 2010–2018 while entangled in a packing band, possibly indicating that sea lions are able to shed packing bands at a higher rate than other materials. The large, non-zero proportion of individuals exhibiting entanglement-related scarring in our record and in other studies [16,20,41] is another testament both to the ability of animals to self-shed entangling materials and to survive even severely wounding entanglements. The prevalence of animals with entanglement scars, the lack of animals stranded dead entangled in packing bands, and observations of animals shedding entangling materials all point to higher entanglement survival than is currently assumed. In this study we did not formally categorize the body condition of entangled individuals, but it was our impression that most were in good condition, indicating the need to evaluate if entanglements cause sub-lethal impacts on individuals. That so many separate lines of evidence point to frequent survival of entangled pinnipeds signals the need to better understand entanglement-related injury and survival rates to be able to account for the impacts of these injuries more accurately within pinniped populations.

While the lack of recorded mortalities due to entanglement in the stranding record and published literature can be somewhat attributed to animals not always dying from entanglement, it is also likely that some affected animals are dying at sea or otherwise away from areas where they might be detected [4,9,62,63]. Entanglement in a large entangling material, such as a trawl netting fragment, has been proven to increase the energy expenditure of affected animals, increase the time they spend at sea, and decrease the depth and duration of foraging dives, all of which could lead to reductions in health or survival and cause them to perish away from the scientific eye [61,63,64]. Internal entanglement injuries from swallowed and embedded hooks are also likely to go undetected and unrecorded by stranding networks particularly when carcasses are in an advanced state of decay. Flashers made up one third of strandings with an identifiable entanglement, a much higher proportion than what was seen in live observations (13.6% Steller, 6.3% California), indicating that individuals with entanglements caused by a swallowed hook could have a higher mortality rate. The presence of flasher entanglements on live individuals only during June–September reinforces that sea lions either quickly shed the gear or die. Most sea lions were in good body condition when observed with a flasher, suggesting it is more likely that they quickly shed the gear, though some animals retain the hook internally after losing the visible flasher. Three animals in the Oregon stranding record had hooks in their stomach and esophagus, but no external signs of entanglement, and one individual was found with a hook in the stomach and the attached flasher wedged in the esophagus, demonstrating that animals impacted by embedded hooks may have sustained severe injuries without showing any observable evidence of entanglement until necropsy [65]. Likewise, animals entangled in derelict fishing gear, such as ghost nets, are unlikely to be discovered until the gear is recovered, so the impact of these entanglement mortalities is likely underestimated [66]. At-sea mortality, internal injuries, and derelict gear are just a few types of entanglement-related mortality unlikely to be accurately documented and included in published entanglement rates.

The type of entangling material can also potentially impact the likelihood of observing an entanglement. If sea lions entangled by a salmon flasher are likely to either shed the gear quickly or die, the window to observe and document that entanglement might be much shorter than for a material more prone to long entanglements, like a packing band. The shape and color of the entangling material could also contribute to the probability that it is observed. Packing bands, rope, and monofilament line all mostly cause neck collar entanglements, but monofilament line, which is thin and usually somewhat translucent, is likely to be quickly embedded in a deep wound, disappearing from view faster than a thicker packing band or rope loop would. Packing bands also have a distinctive fraying pattern which causes thin curly strands to be visible above the edges of a deep wound where the band itself is otherwise invisible, making them much more likely to be identified than a material without such clear identifying features. For the most part, it was impossible to identify the entangling material in cases of severe entanglement wounds because the material was embedded so deeply in the flesh, and therefore also impossible to make any conclusions about which materials might be associated with the most severe wounds or highest potential risk of mortality to the affected individual. Additionally, only the most severe, deep, wide wounds are likely to create lasting and readily observable scars, meaning certain entangling materials are better represented among scarring rates than others. This complicates the search for the most damaging entangling materials on which to focus targeted mitigation and forces any management efforts to rely on other metrics of impact, such as the prevalence of an entangling material within the population in question. Further studies that track the fate of individually identifiable entangled individuals would help clarify important questions about scar healing rates and time to death or shedding that are crucial for understanding the full long- and short-term impact of entanglement on individuals and populations.

The age, size, and foraging experience of the sea lion may dictate the materials they become entangled in, and therefore the outcome and observability of the entanglement [9,67,68]. The high proportion of entangled Steller juveniles exhibiting flashers and rubber bands may be a function of their age: rubber bands may be too small to entangle a large adult, and flasher entanglement is a sign of a risky foraging behavior—depredating salmon troll fisheries. The small number of unidentifiable entangling materials on juveniles may be because of their smaller size, which causes the material to sit on the surface of the skin where it can be easily identified. This may also explain the large number of unidentifiable entangling materials on adult males, whose considerable seasonal growth [69] could have caused entanglements to bury deep into the flesh where they are not readily observed [61]. Age and body size therefore impact both the entangling materials an individual is likely to encounter, and the severity of the wound caused by that entanglement.

Entanglement may also have an impact on pinniped life history and population dynamics. Most California sea lions migrate away from our survey area to their breeding grounds to the south during June and July, but the few animals that stayed in our survey area during those months exhibited a much higher entanglement rate than in other months, largely driving the high overall rate of entanglement seen for California sea lions (Fig 5). In our study area, it is possible that entangled male California sea lions observed in the summer months chose not to migrate to their breeding grounds due to compromised body condition caused by entanglement, which would likely also compromise their ability to establish and hold a breeding territory. This confirms that counts of entangled individuals taken from non-rookery sites only during the summer months might be useful as an index of change in entanglement occurrence but cannot be used to predict population-wide or annual average entanglement rates without other sources of data. Even for individuals that did arrive at their breeding grounds, entanglement could impact their reproductive success. In Alaska, entangled nursing female northern fur seals spent longer at sea, weaned smaller pups, and abandoned their pups more frequently than unentangled females [61,70]. However, records of three entangled female California sea lions successfully weaning pups in Los Islotes, Baja California [16] and our observation of at least one entangled Steller sea lion female nursing a pup demonstrate that the impacts of entanglement on all aspects of pinniped population dynamics, especially long-term impacts, are poorly understood.

Entanglement rates also appear to be impacted by the availability and distribution of entangling materials in the immediate environment [4,9]. In our survey area, the occurrence of packing bands in beach surveys was positively correlated with the proportion of entangled individuals exhibiting packing bands. A similar relationship has been observed in Hawaiian monk seals, which frequently haul out on top of beached debris and therefore experience higher entanglement risk when more debris is present on the beach [71], and with northern fur seal pups which show higher rates of entanglement in areas on St. Paul Island, Alaska with higher concentrations of debris in the nearshore [68]. It is likely that both basin-wide circulation patterns and nearshore currents play a role in the concentration of entangling materials and therefore the distribution of entanglement hot spots. Studies have shown that warm anomaly ocean conditions, usually associated with an El Niño event, can cause changes to the distribution of marine debris, fishing effort, and pinniped prey items, all of which can impact rates of entanglement [14,15,38]. In summer 2014, high sea surface temperatures associated with the warm anomaly referred to as “the Blob” reached the coast, causing the shortest upwelling season for the northern California Current on record [72], the impacts of which were seen well into 2016 [73]. Both California and Steller sea lions exhibited high rates of entanglement in our study area in 2014 and 2015, and 2014–2016 were also years of elevated large whale entanglements in the area [17,74]. It is possible that these anomalous ocean conditions changed the distribution of fishing effort, entangling materials, and prey items important to cetaceans and pinnipeds, causing habitat compression and contributing to the high levels of entanglement seen for both taxa. Entanglement rates therefore seem to be driven somewhat by normal ocean currents and abnormal ocean conditions. However, the way that ocean conditions impact entanglements may depend on the type of entangling material, as actively fished and derelict gear are more likely to be impacted by conditions that shift fishing effort, prey distributions, and sea lion abundance, while marine debris is more likely to be linked to conditions that directly change currents and circulation.

Our study showed high haulout abundance increase rates in Steller and California sea lions in Washington despite high entanglement rates, suggesting that entanglement is not an issue that requires immediate conservation attention in this area. However, entanglement is still a significant welfare issue for individual sea lions. Considering that most entanglements are caused by humans, through the creation of marine debris, derelict fishing gear, or direct fishery interactions [4] (except for animals collared by penguin skins [30,31,59]), it becomes a matter of good stewardship to reduce the negative impact on sea lion welfare. The good news is that human-caused entanglements can be addressed through changes in human behavior. For entanglements caused by actively fished gear, outreach and education paired with deterrence strategies may prove effective, while marine debris requires tackling pollution sources or redesigning offending materials. In New Zealand and South Georgia, campaigns to encourage fishers to cut packing bands before disposal led to declines in packing band entanglements [16,75]. However, in Australia, large-scale efforts by the government and local fishers to reduce entanglement failed to prevent entanglement rates from continuing to increase [36]. Page et al. (2004) proposed that the debris could originate from areas outside of Australian waters and away from local fishing grounds, making national legislation ineffective at addressing the trans-boundary issue. A similar situation could complicate entanglement prevention efforts in northern Washington because of the close proximity to the Canadian border and the presence of large basin-wide currents just offshore. Page et al. (2004) also commented that laws that fall short of mandating the use of redesigned materials to prevent entanglement risk, such as biodegradable packing bands, may fail to cause an effective change in observed entanglement rates. Similarly, while deterrents exist or are in development that could prevent animals from interacting with various types of actively fished gear [76,77], it can be a challenge to find a solution that balances effectiveness, cost, and reduction of potential harm to the ecosystem [78–80]. While preventing entanglements altogether is likely an impossible task, small actions such as encouraging fishers to cut packing bands could decrease the impact of entanglement on the welfare of local pinniped species.
